# Fast-to-Slow Transition of Skeletal Muscle Contractile Function and Corresponding Changes in Myosin Heavy and Light Chain Formation in the R6/2 Mouse Model of Huntington’s Disease

**DOI:** 10.1371/journal.pone.0166106

**Published:** 2016-11-07

**Authors:** Tanja Hering, Peter Braubach, G. Bernhard Landwehrmeyer, Katrin S. Lindenberg, Werner Melzer

**Affiliations:** 1 Institute of Applied Physiology, Ulm University, Ulm, Germany; 2 Department of Neurology, Ulm University, Ulm, Germany; 3 Institute of Pathology, Hannover Medical School, Hannover, Germany; University of Debrecen, HUNGARY

## Abstract

Huntington´s disease (HD) is a hereditary neurodegenerative disease resulting from an expanded polyglutamine sequence (poly-Q) in the protein huntingtin (HTT). Various studies report atrophy and metabolic pathology of skeletal muscle in HD and suggest as part of the process a fast-to-slow fiber type transition that may be caused by the pathological changes in central motor control or/and by mutant HTT in the muscle tissue itself. To investigate muscle pathology in HD, we used R6/2 mice, a common animal model for a rapidly progressing variant of the disease expressing exon 1 of the mutant human gene. We investigated alterations in the extensor digitorum longus (EDL), a typical fast-twitch muscle, and the soleus (SOL), a slow-twitch muscle. We focussed on mechanographic measurements of excised muscles using single and repetitive electrical stimulation and on the expression of the various myosin isoforms (heavy and light chains) using dodecyl sulfate polyacrylamide gel electrophoresis (SDS-PAGE) of whole muscle and single fiber preparations. In EDL of R6/2, the functional tests showed a left shift of the force-frequency relation and decrease in specific force. Moreover, the estimated relative contribution of the fastest myosin isoform MyHC IIb decreased, whereas the contribution of the slower MyHC IIx isoform increased. An additional change occurred in the alkali MyLC forms showing a decrease in 3f and an increase in 1f level. In SOL, a shift from fast MyHC IIa to the slow isoform I was detectable in male R6/2 mice only, and there was no evidence of isoform interconversion in the MyLC pattern. These alterations point to a partial remodeling of the contractile apparatus of R6/2 mice towards a slower contractile phenotype, predominantly in fast glycolytic fibers.

## Introduction

Huntington’s disease (HD) is a genetic neuropathy associated with severe motoric dysfunction [1, 2]. HD is of autosomal dominant inheritance and results from an expanded cytosine-adenine-guanine triplet repeat (CAG)_n_ in exon 1 of the IT15 gene (*HTT*) leading to an elongated polyglutamine (polyQ) stretch in the N-terminal region of the protein huntingtin (HTT). The pathology in HD is thought to result mainly from a gain of function caused by toxic polyglutamine-containing fragments that are capable of forming protein aggregates [3]. In the brain, neurons of the striatum and cortex are predominantly affected [4, 5]. The pathomechanism likely involves a combination of effects, for instance on vesicular transport, endocytosis, the ubiquitin-proteasome system and transcription [6, 7]. A number of studies indicate mitochondrial dysfunction as an important contributor to the pathology [8–14]. In addition several results point to altered Ca^2+^ homeostasis and excitotoxicity in affected neurons [15, 16].

HTT is ubiquitously expressed both in the central nervous system and in peripheral organs [17–20] leading to tissue-specific changes. The most obvious clinical alteration in peripheral tissues in HD are muscle weakness and wasting [21, 22], probably caused by both neuronal and muscle-intrinsic mHTT-induced changes [23–27] [28][29, 30].

Muscle as an extensive and easily accessible excitable tissue may, therefore, provide clues regarding mechanisms of the disease development and has been considered as a potential biomarker for its progression and for monitoring therapeutic interventions [31]. Among the animal models, the R6/2 mouse used in the present investigation has been one of the most frequently studied. It originated from an insertion of the N-terminal fragment (exon 1) of the mutant human huntingtin gene (*mHTT*, with 144 CAG repeats) [32]. Widespread mHTT-positive inclusion formation in several neuronal and non-neuronal tissues which include muscle have been observed in R6/2 mice [19, 24, 25, 27, 29]. Several studies have addressed skeletal muscle function in these mice and identified higher sensitivity of mitochondria for Ca^2+^-induced permeability transition [33], alterations in synaptic transmission and in Na^+^, K^+^ and Cl^-^ channels [34, 35] and compromised depolarization-triggered Ca^2+^ release and removal activity and contraction [30, 36].

RNA microarray analysis of the pattern of gene expression in skeletal muscle of human HD patients and R6/2 mice suggested a “progressive loss of fast glycolytic fibers and concomitant gain in slow fibers” [31]. The four main types of muscle fibers found in mouse skeletal muscle, I, IIA, IIX (or IID) and IIB, are characterized by their expression of the four myosin heavy chain (MyHC) isoforms I, IIa, IIx (or IId) and IIb, respectively. I and IIA are oxidative and IIX/D and IIB glycolytic fibers with I representing the slowest and IIB the fastest contracting fiber type [37, 38]. Each myosin heavy chain is associated with two light chains (MyLC), termed essential (or alkali) and regulatory (or phosphorylatable) light chain, respectively. Light chains are present in mouse muscle in 5 different isoforms (1f, 2f, 3f, 1s and 2s). Here 1 and 3 indicate essential and 2 regulatory isoforms and the letters indicate their predominant presence in fast glycolytic (f) and slow oxidative (s) muscle fiber types, respectively. The hypothesis of a fast to slow transition would imply that the expression pattern of myosin isoforms changes [37, 39]. Mielcarek et al. [30] report significant transcriptional upregulation of Myh7, the gene encoding the slow isoform MyHCI, in several fast twitch muscles. In contrast, determining protein alterations in R6/2 interosseus muscle (containing predominantly fast type IIA fibers), our group found several alterations in the MyLC protein pattern but none in MyHC isoforms that would have pointed to a significant fast to slow fiber type transition [36]. Therefore, our aim in this study was to specifically investigate the fast glycolytic extensor digitorum longus muscle (EDL) in comparison to the slow oxidative soleus (SOL) muscle to determine contractile properties and to quantify changes in muscle fiber type by determining relative alterations of the various myosin isoforms using electrophoretic protein separation and immunostaining.

## Materials and Methods

### Ethics statement

All experimental procedures performed on mice were in accordance with German animal protection laws and conducted under the project licence (C/0.113) of the Institutional Animal Care and Use Committee of Ulm University (Tierforschungszentrum, Universität Ulm and the Regierungspräsidium Tübingen) that specifically approved this study.

### Experimental animals

R6/2 mice [32] (B6CBA-Tg (HDexon1)62Gpb/1J x C57BL6J/CBA/caF1; The Jackson Laboratory) with 190 ± 10 CAG repeats and wild-type (WT) littermates were kept at a 12 hour light-dark cycle with unrestricted access to food and water. Animal handling was in agreement with the regulations of the local animal welfare committee. Genotype and CAG length were determined from tail biopsies according to Mangiarini et al [32]. Mice, showing weight loss (on average 20–25% compared to WT), muscle atrophy and clasping, were sacrificed at the age of 12 to 13 weeks by CO_2_ application and rapid cervical dislocation.

### Contraction measurements

Extensor digitorum longus (EDL) and soleus (SOL) muscles were dissected from the hind limbs of mice and transferred to carbogen-bubbled Ringer’s solution [40]. Muscles were vertically mounted in a temperature-controlled test chamber and connected to a force transducer (FT03, Grass Instruments, Quincy USA). After stretching the muscle to optimal length, single or repetitive contractions were elicited with supra-maximal electric current pulses of 1 ms duration passed through platinum electrodes positioned near the muscle and recorded using a bridge amplifier and data acquisition system (Digidata 1200, Axon Instruments) controlled by custom-made software. Cross sectional area was calculated from muscle diameter under the assumption of a circular cross-section. In force-frequency experiments contractile forces were normalized to the force observed during the 125 Hz (EDL) and 100 Hz (SOL) tetani.

### Myosin heavy chain analysis

Muscle specimens were immersed in a solution consisting of 50% glycerol and 50% Krebs-Ringer’s solution, then rapidly frozen in liquid nitrogen and stored at -80°C. For the following MyHC determination always one muscle (SOL and EDL) was used, while the second SOL and EDL muscle was used for MyLC determination. After re-thawing a protein extraction according to Singh et al. [41] was performed. Briefly, one SOL and EDL per mouse was subjected to a protein extraction buffer containing (in mM) 300 NaCl, 100 NaH_2_PO_4_, 50 Na_2_HPO_4_, 10 Na_4_P_2_O_7_, 1 MgCl_2_, 10 EDTA, 1.4 2-mercapto-ethanol. They were cut into small pieces and vortexed for 10 s. A repetitive freezing (liquid nitrogen) and re-thawing procedure improved protein extraction and was followed by 10 min centrifugation at 10,000 rpm. For the MyHC determination in single fibers, freshly dissected muscles were incubated under permanent shaking for 45 minutes at 35°C in 3 ml Ringer’s solution containing 2 mg/ml collagenase. The digestion was stopped with PBS (phosphate-buffered saline; 20012; Invitrogen GmbH; Darmstadt) containing 10% FCS. After thorough washing with Krebs-Ringer’s solution and transfer to a depolarizing buffer (58 mM TES, 7.8 mM MgCl_2_, 50 mM EGTA, 1 mM KH_2_PO_4_, 6.2 mM Na_2_ATP, pH 7.1) intact single fibers were randomly selected and used for protein extraction as described by Tikunov et al. [42]. Total protein in the supernatant was estimated using Bradford’s method. A Mini-Gel system (Bio-Rad, Munich, Germany) was used for SDS-PAGE. The gels (8% polyacrylamide) were loaded with 5 μg extracted protein from whole muscle or total single fiber lysate [38] and stained with Roti®-Blue (Carl Roth; Karlsruhe, Germany) and the protein bands in the scanned gels were integrated using the software ImageJ (National Institutes of Health). Quantitative Western Blot analysis to identify the stained MyHC isoforms was performed as described by Braubach et al. [36].

### Myosin light chain analysis

To avoid a contamination by other proteins of similar molecular weight, a myosin extraction method [43] was used when studying MyLC isoforms. For this, the second SOL and EDL of each mouse was used. Separation of 25 μg myosin extraction was performed using 12% polyacrylamide gels.

### Statistics

Statistical calculations were carried out using R 3.1 [44] running under Ubuntu Linux 15.04 and Microsoft Windows 7. Data are presented as mean value ± SEM (n = number of values), count data as relative frequencies and binomial proportion confidence intervals (95% CI) using Wilson’s method. Group means were compared by Student’s two-sided t-test, distributions by the chi-squared test. In figures significant differences are marked as follows: *: p < 0.05, **: p < 0.01, ***: p < 0.001.

## Results

### Contraction measurements

**Fig 1:**
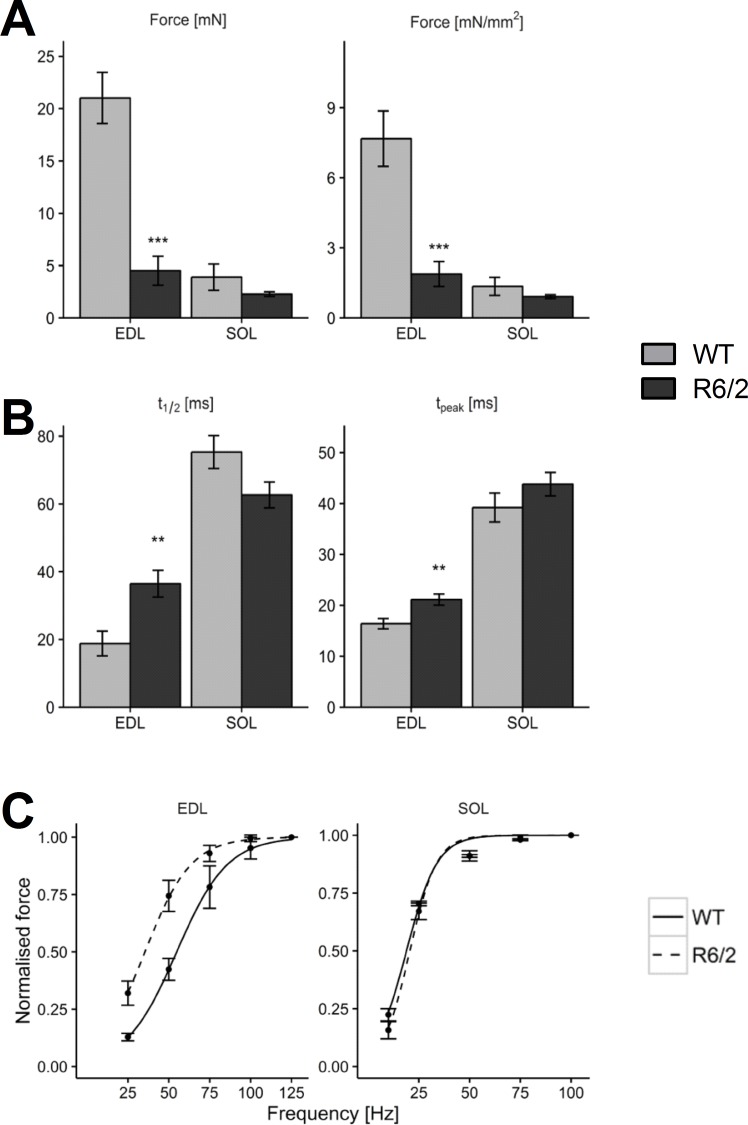
Lower force and slowed kinetics of contraction in R6/2. (A) Mean twitch force (left panel) and specific force (i.e. normalized by cross sectional area; right panel) compared in EDL and SOL muscles of WT (n = 15 and n = 15, respectively) and R6/2 (n = 10 and n = 10, respectively). (B) Comparison of half time of relaxation (t_1/2_, left panel) and time to peak (t_peak_, right panel). (C) Comparison of force frequency relations. Error bars indicate SEM.

EDL and SOL differ strongly in their fiber type pattern. EDL is composed of mainly glycolytic fibers, whereas SOL contains large amounts of fast (type IIa) and slow (type I) oxidative fibers [45] and exhibits a slower twitch contraction [40]. On average, isolated muscles of R6/2 mice showed a reduced weight compared to their age-matched WT counterparts (6.67 vs. 12.1 mg for EDL and 6.27 vs. 8.00 mg for SOL) and smaller estimated cross-sectional area (2.25 vs. 2.84 mm^2^ for EDL and 2.49 vs. 2.72 mm^2^ for SOL). To characterize the contractile properties, isometric force of the muscles was recorded in response to single or repetitive electrical shocks of 1 ms duration. Similar to Mielcarek and coworkers [30], it was found that contractile force in R6/2 EDL was reduced. Peak twitch and tetanic force of the EDL were significantly smaller both in absolute size and when normalized for cross-sectional area (Twitch: 1.88 vs. 7.67 mN/mm^2^; **Fig 1A**); tetanus: 24.2 vs. 59.6 mN/mm^2^**)**. Therefore, reduced force is not a mere consequence of smaller muscle size. In the SOL, which produced lower specific force than EDL the changes were considerably smaller and not significant (Twitch: 0.92 vs. 1.35 mN/mm^2^; tetanus: 39.4 vs. 41.4 mN/mm^2^).

To determine force-frequency relations, incomplete and fused tetani (lasting 350 ms) were measured in EDL at frequencies of 25, 50, 75, 100 and 125 Hz. In SOL, frequencies of 10, 25, 50, 75, 90, and 100 Hz were applied. **Fig 1C** shows mean maximum force as a function of stimulation frequency normalized to the value at the highest frequency, respectively. The normalized force-frequency relations of R6/2 and WT were almost identical in SOL whereas in EDL a clear shift to the lower frequencies can be noticed for R6/2.

Consistent with these observations, kinetic parameters of single twitch responses showed muscle type specific changes: A significant increase in the time to the peak of the twitch (t_peak_) and the half-time of relaxation (t_1/2_) was observed in EDL (by 128% and 190%, respectively), but no corresponding alteration could be noticed in SOL (**Fig 1B**). Data of the contraction experiments are summarized in **Table 1**. For single-twitch measurements duplicate measurements with replication in both muscles (left and right) were performed in 15 WT and 10 R6/2 animals. For force-frequency analysis 15 muscles of 15 WT and 10 muscles of 10 R6/2 animals were analysed.

**Table 1:** Summary of muscle contractile properties.

Muscle	Parameter	WT ± SEM	n (Animals)	R6/2 ± SEM	n (Animals)	p value
EDL	Weight	12.11 ± 1.09 mg	15 (duplicate)	6.67 ± 0.36 mg	10 (duplicate)	< 0.001
	CSA	2.25 ± 0.10 mm^2^	15 (duplicate)	2.84 ± 0.11 mm^2^	10 (duplicate)	< 0.001
	Specific Force (Twitch)	1.88 ± 0.53 mN/ mm^2^	15 (duplicate)	7.67 ± 1.18 mN/ mm^2^	10 (duplicate)	< 0.001
	Specific Force (Tetanus)	24.18 ± 3.50 mN/mm^2^	14 (single)	59.63 ± 10.82 mN/mm^2^	5 (single)	0.02
SOL	Weight	6.27 ± 0.29 mg	15 (duplicate)	8.00 ± 0.52 mg	10 (duplicate)	0.01
	CSA	2.49 ± 0.05 mm²	15 (duplicate)	2.72 ± 0.09 mm²	10 (duplicate)	0.04
	Specific Force (Twitch)	0.92 ± 0.08 mN/mm^2^	15 (duplicate)	1.35 ± 0.38 mN/mm^2^	10 (duplicate)	0.29
	Specific Force (Tetanus)	39.38 ± 3.19 mN/ mm^2^	15 (single)	41.41 ± 6.41 mN/ mm^2^	7 (single)	0.78

Values are reported as mean ± SEM. n is the number of animals. Single twitch measurements are in duplicates (left and right muscle of each animal), tetanus measurements as single muscle. CSA: cross sectional area.

An important determinant of muscle contraction kinetics is the cross-bridge interaction with actin of the motor protein myosin. Myosin isoforms are also important indicators of muscle plasticity and fiber type changes [37]. To investigate whether the observed kinetic alterations go in parallel with alterations in motor proteins, we used SDS-PAGE to quantify relative changes in MyHC and MyLC isoform expression.

### Myosin heavy chain quantification

To check whether the myosin determination depends on the concentration of protein used in the electrophoresis, we first performed a series of runs with different quantities of applied total protein. **S1 Fig** shows stained example gels for EDL and SOL, respectively (both WT), exploring the range from 0.5 to 8 μg of applied protein. The figure shows that EDL expresses only fast isoforms IIx and IIb, whereas SOL expresses IIa and I and only traces of IIx. The lower panels in **S1 Fig** demonstrate that in the investigated range the fractional contribution of each isoform is independent of the starting quantity of muscle protein used. In the following experiments, 5 μg of protein was applied for the comparison of MyHC expression in WT and R6/2 muscle.

**Fig 2:**
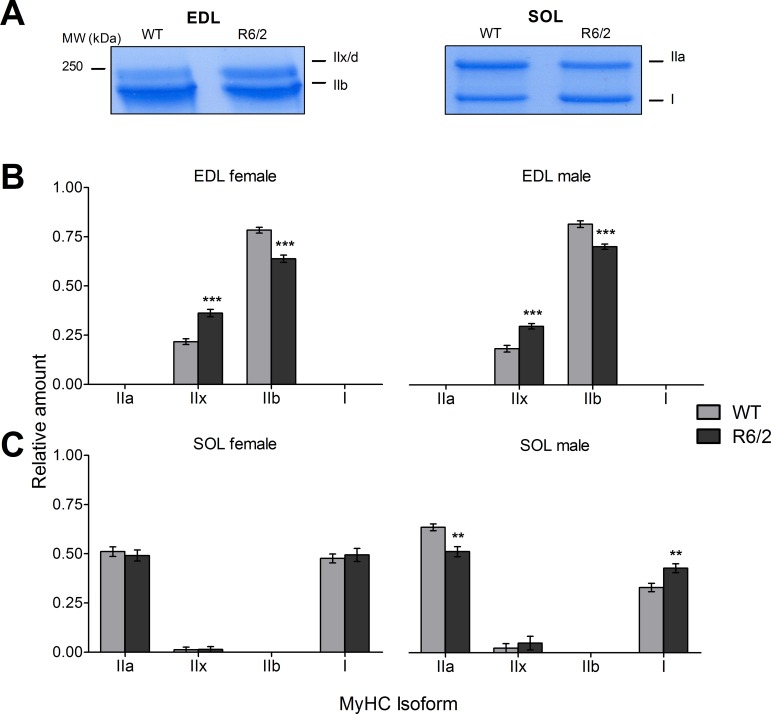
Relative content of myosin heavy chain isoforms in WT and R6/2 muscle. (A) Examples showing sections of Roti®-Blue-stained gels exhibiting MyHC bands that were evaluated in the analysis. See also **S1 Fig**. (B) Relative amounts of the indicated MyHC isoforms in EDL of WT and R6/2, respectively, presented separately as results from female (left panel) and male specimen (right panel). (C) Relative amounts of the indicated MyHC isoforms in SOL of WT and R6/2, respectively, presented separately as results from female (left panel) and male (right panel) specimen. Error bars indicate SEM. n = 11 to 13 mice.

**Fig 2A** shows the MyHC bands of representative gels (EDL left, SOL right; WT and R6/2, respectively). **Fig 2B and 2C** compare relative amounts of isoforms using mean data from equal numbers (11 male and 12 female) of WT and age-matched R6/2 mice, respectively. Values are summarized in **Table 2**. The EDL (**Fig 2B**) showed a highly significant decrease in the relative amount of MyHC IIb (by 13.8% in males and 18.5% in females) and a corresponding increase in MyHC IIx. While there was no significant gender difference noticeable in EDL, results from the SOL differed in male and female mice (**Fig 2B, Table 2**). Alterations were only observed in male mice. Here, a decrease (by 19.4%) in MyHC IIa and corresponding increase in MyHC I could be noticed. Traces of isoform IIx were also noticable in some SOL muscles (1 of 11 male mice and 1 of 12 female WT mice and 3 of 11 male R6/2 mice and 1 of 12 female R6/2 mice) but the slight increase in R6/2 was not significant.

**Table 2:** Summary of myosin heavy chain (MyHC) distribution (fractional content) in EDL and SOL muscles of male and female R6/2 mice and WT controls.

	MyHC		WT ± SEM	n (WT mice)	R6/2 ± SEM	n (R6/2 mice)	Difference (R6/2-WT)	Change (%)	p value
**Male mice**	SOL	IIa	0.642 ± 0.017	12	0.518± 0.026	10	-0.125	-19.40%	0.001
		IIx/d	0.024 ± 0.024		0.049 ± 0.035		0.026	107.76%	0.55
		IIb	-		-		-	-	-
		I	0.334 ± 0.021		0.434± 0.023		0.099	29.77%	0.005
	EDL	IIa	-	12	-	10	-	-	-
		IIx/d	0.185± 0.017		0.297 ± 0.014		0.112	60.79%	<0.001
		IIb	0.815± 0.017		0.703 ± 0.014		-0.112	-13.78%	<0.001
		I	-		-		-	-	-
**Female mice**	SOL	IIa	0.512 ± 0.025	12	0.490± 0.028	12	-0.02	-4.30%	0.56
		IIx/d	0.013± 0.013		0.014 ± 0.014		0.002	11.98%	0.94
		IIb	-		-		-	-	-
		I	0.475 ± 0.023		0.49± 0.034		0.021	4.32%	0.62
	EDL	IIa	-	12	-	12	-	-	-
		IIx/d	0.217± 0.015		0.362 ± 0.018		0.145	66.91%	<0.001
		IIb	0.783± 0.015		0.638 ± 0.018		-0.145	-18.55%	<0.001
		I	-		-		-	-	-

Values presented as mean ± SEM.

### Myosin determination in single muscle fibers

In addition to the pure fiber types corresponding to the four MyHC variants, mixed fibers expressing different heavy chain isoforms have been identified [46]. We also carried out SDS-PAGE and Western blotting using single muscle fibers of the EDL to receive a more detailed picture of the changes in myosin heavy chain concentration observed in whole EDL of R6/2 compared to WT mice.

**Fig 3:**
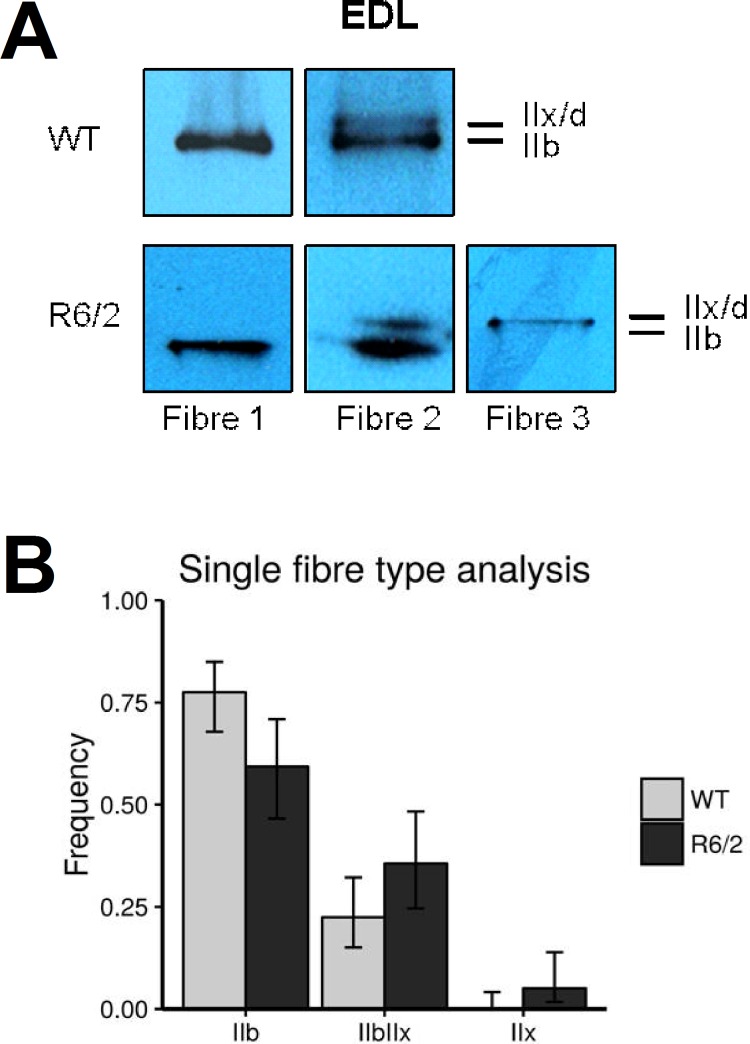
MyHC determination in single muscle fibers. Muscle fiber composition for EDL was assessed on a single fiber basis. For this MyHC were extracted from 6 to 15 randomly selected, intact fibers per muscle sample. (A) Examples showing Roti®-Blue-stained MyHC bands from selected single fiber SDS PAGE gels. Fibers were classified as Type IIB and IIX when a single band could be detected and as mixed IIB/X when both bands were detectable, irrespective of staining intensity. (B) Relative contribution (fractional number) of pooled fibers (59 fibers of 5 R6/2 mice and 89 fibers of 7 WT mice) exhibiting expression of MyHC IIb, IIx or both. Distributions of fibers amongst the types IIB, IIX and mixed type IIB/X differed significantly between WT and R6/2 mice (p = 0.02) indicating a different muscle fiber composition of R6/2 EDL muscle with more mixed-type fibers. Bars show relative frequencies; error bars show confidence intervals for binomial proportions (i.e. fibers of respective type vs. all other fibers) (95% CI).

A total of 59 fibers from 5 R6/2 mice and 89 fibers from 7 WT mice were analyzed. Western blot examples are shown in **Fig 3A**). We found fibers that expressed MyHC isoforms IIb, IIx and both. However, the distribution of fiber types differed significantly between R6/2 and WT muscles (p = 0.02). The diagram in **Fig 3B** shows the relative contribution of each of the three groups of fibers. In WT mice, 78% (95% confidence interval (CI): 68%–85%) of the fibers belonged to the pure IIB-type (i.e. contained isoform IIb exclusively), most of the remaining fibers expressed both isoforms (IIb and IIx; 22%; 95% CI: 15%–32%). In R6/2 mice a higher fraction (36%; 95% CI: 25%–48%) of mixed fibers was found and a small fraction of pure IIX fibers (3 in 59, respectively 5%; 95% CI 2%–14%). Correspondingly, the fraction of pure IIB fibers decreased to 59% (CI: 46%–71%). These observations are consistent with the results from whole EDL and indicate the additional expression of the MyHC IIx isoform in formerly pure IIB fibers and perhaps the disappearance of IIb in some mixed fibers. Overall, this corresponds to a modest transition to a slower contractile apparatus.

### Myosin light chain quantification

Although the main characteristics of the mechanical performance of a muscle fiber is determined by their MyHC content, the small myosin subunits (myosin light chains, MyLC) appear to be modulators of the heavy chain motor performance [47–49]. Therefore, we also investigated possible changes of their relative expression in R6/2 compared to WT muscle. To account for their lower molecular weight, we separated these proteins using gels of higher density (12% compared to the 8% used for the MyHCs). Again, we first varied the amount of protein that was used in the SDS PAGE runs to verify that results were not dependent on protein concentration (**S2 Fig).** The panels A1 and B1 of **S2 Fig** show examples of complete gels demonstrating the MyLC band pattern at different starting amounts of applied protein ranging from 5 to 30 μg. Panels A2 and B2 show the relative contributions of the different isoforms evaluated as the means from n = 9 different gels each. In the EDL (A), the regulatory isoform MyLC2f is most abundant followed by the alkali isoform MyLC1f. Considerably weaker is the band of MyLC3f, the second alkali isoform expressed in fast muscle. The slow alkali and regulatory isoforms (MyLC1s and MyLC2s, respectively) were not detectable in the EDL gels. In contrast, all five myosin light chains were found in SOL with MyLC1s producing the most pronounced signal and MyLC3f by far the least intense band.

**Fig 4:**
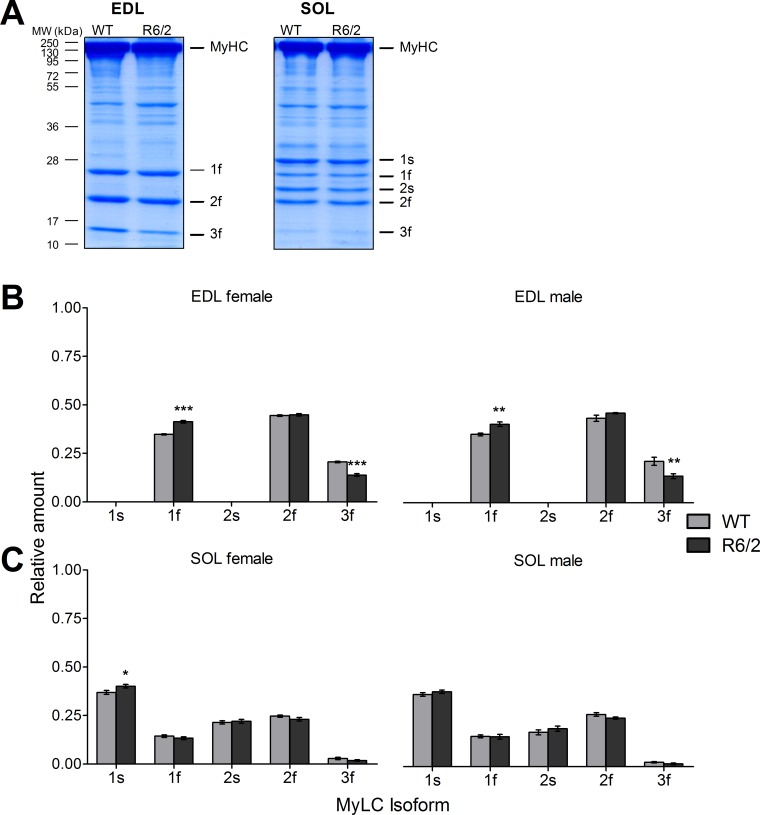
Relative content of myosin light chain isoforms in WT and R6/2 muscle. (A) Examples showing Roti®-Blue-stained gels exhibiting MyLC bands that were evaluated in the analysis. See also **S2 Fig**. (B) Relative amounts of the indicated MyLC isoforms in EDL of WT and R6/2, respectively, separated in results from female (left panel) and male specimen (right panel). (C) Relative amounts of the indicated MyLC isoforms in SOL of WT and R6/2, respectively, separated in results from female (left panel) and male (right panel) specimen. Error bars indicate SEM. n = 10 to 12 mice.

When comparing R6/2 with WT (**Fig 4**) we used 25 μg of applied protein in the electrophoresis. **Fig 4A** shows representative examples of stained gels for EDL (left) and SOL (right), respectively. In the analysis of all muscles, significant changes could only be recognized in EDL (**Fig 4B)**. Here, the relative contribution of MyLC3f decreased, whereas that of MyLC1f increased both in male and female mice (see also **Table 3**). MyLC2f showed no significant alterations. In SOL the pattern remained essentially unchanged (except for a small significant increase in 1s in females only).

**Table 3:** Summary of myosin light chain (MyLC) distribution (fractional content) in EDL and SOL muscles of male and female R6/2 mice and WT controls.

		MyLC	WT ± SEM	n (WT mice)	R6/2 ± SEM	n (R6/2 mice)	Difference (R6/2-WT)	Change (%)	p value
**Male mice**	SOL	1s	0.371 ± 0.009	10	0.387 ± 0.009	12	0.016	4.17%	0.25
		1f	0.156 ± 0.007		0.154 ± 0.013		-0.002	-1.22%	0.90
		2s	0.182 ± 0.012		0.196 ± 0.014		0.014	7.60%	0.46
		2f	0.269 ± 0.008		0.250 ± 0.006		-0.019	-7.14%	0.07
		3f	0.022 ± 0.014		0.014 ± 0.004		-0.008	-36.72%	0.14
	EDL	1s	-	10	-	10	-	-	-
		1f	0.352 ± 0.007		0.404 ± 0.012		0.057	16.14%	<0.001
		2s	-		-		-	-	-
		2f	0.435 ± 0.016		0.461 ± 0.003		0.020	4.51%	0.24
		3f	0.213 ± 0.021		0.146 ± 0.012		-0.008	-36.20%	0.003
**Female mice**	SOL	1s	0.369 ± 0.010	10	0.401 ± 0.009	12	0.031	8.36%	0.03
		1f	0.142 ± 0.006		0.133 ± 0.008		-0.029	-6.45%	0.36
		2s	0.214 ± 0.007		0.220 ± 0.010		0.005	2.25%	0.70
		2f	0.247 ± 0.005		0.230 ± 0.009		-0.017	-6.77%	0.13
		3f	0.028 ± 0.005		0.018 ± 0.004		-0.010	-34.90%	0.16
	EDL	1s	-	10	-	12	-	-	-
		1f	0.349 ± 0.003		0.413 ± 0.007		0.064	12.78%	<0.001
		2s	-		-		-	-	-
		2f	0.446 ± 0.004		0.449 ± 0.005		0.003	1.59%	0.67
		3f	0.205 ± 0.004		0.138 ± 0.007		-0.067	-28.62%	<0.001

Values presented as mean ± SEM.

In summary, clear changes in isometric force and twitch kinetics correlating unambiguously with transitions to slower isoforms of myosin heavy and light chains were restricted to EDL muscle in R6/2 mice.

## Discussion

### HD and muscle pathology

The pathology of Huntington’s Disease has largely been attributed to dysfunction of the central nervous system. However, there is growing evidence that peripheral tissues are affected, too, caused by the ubiquitous expression of huntingtin [17–19, 27, 29, 50, 51]. Skeletal muscle defects in HD are well documented [21, 22, 33, 52–54] and first signs of reduced muscle performance have been detected before neuronal symptoms became manifest [21]. It is still unresolved whether these changes result from muscle-intrinsic mutant huntingtin or are the consequence of defects in the motoric nervous system. A recent study in two murine models of HD (R6/2 and HdhQ150) suggested that both scenarios contribute [30]. They observed up-regulation of transcripts for proteins involved in slow-type contractile activation and corresponding metabolic changes and identified alterations in regulatory pathways possibly underlying these changes. These results supported the notion of a fast to slow muscle fiber transition that had been suggested earlier [31] and pointed to denervation and loss of functional motor units as part of the pathology. Myosin isoforms as established indicators of fiber type [37] have not been quantified at the protein level in HD muscle. Therefore, the present study focused on this aspect in comparison with contractile function using fast and slow R6/2 skeletal muscles.

### Contraction and MyHC changes in R6/2 EDL

Our results support the notion of muscle remodeling to a slower phenotype proposed by others [31, 34]. In accord with Mielcarek et al. [30] a clear alteration to slower contraction kinetics could be observed in the EDL, a muscle that contains only fast twitch, glycolytic muscle fibers and expresses the fast myosin isoforms IIb and IIx/d. In spite of the significant slowing of twitch contraction, myosin isoforms that are typical for slow twitch fibers did not appear in R6/2 EDL. Instead, the relative contribution of the two fast isoforms was found to be altered. The single fiber analysis suggests that this occurs through a complete downregulation of IIb in a fraction of the fibers turning them from pure IIB type to a mixed type IIB/X and to loss of IIb isoforms in some IIB/X fibers turning them into a pure IIX type. MyHC isoform IIx/d exhibits a lower rate of cross-bridge cycling than IIb [55]. Therefore, this isoform switch would indeed qualitatively predict slower isometric twitch contractions.

### MyLC changes in R6/2 EDL

In addition to MyHC, we found a change in MyLC expression in the EDL. Myosin light chains are thought to play a role in stabilizing the alpha-helical neck of the globular myosin head that acts as a lever arm of the motor protein and to fine-tune actomyosin cross bridge cycling [56, 57]. Two functionally different forms are distinguished (MyLC1/3 and 2), termed essential or alkali and regulatory or phosphorylatable light chains, respectively [39]. The regulatory light chains (MyLC2s and MyLC2f) are targets of specific kinases. In smooth muscle, the Ca^2+^-dependent phosphorylation of the regulatory light chains is the mechanism that turns on cross bridge cycling. In skeletal muscle phosphorylation of the regulatory light chains modulates contraction during repetitive activation [58]. mRNA of the phosphorylatable fast myosin light chain was found in smaller amounts in R6/2 than in WT muscle [31]. At the protein level we could not confirm a significant change. Instead the relative expression of alkali isoform 3f decreased whereas 1f increased. MyLC1f and MyLC3f, predominantly expressed in fast fibers, are splice variants originating from the same gene [59]. MyLC1f contains a long extension that is missing in 3f and provides an actin binding site in addition to the globular myosin heads [60]. Probably this is the reason that shortening velocity is reduced by MyLC1f. Unloaded shortening velocity has been reported to be proportional to the relative content of MyLC3f in IIA, IIX and IIB fibers with the sensitivity of fibers to this modulation increasing in the same order [61]. Therefore, our findings of a MyLC3f to MyLC1f transition would also be consistent with slower contraction kinetics in R6/2 EDL.

### Contraction and myosin isoform expression in R6/2 soleus

In contrast to the EDL, functional changes in soleus muscle were much smaller. Neither force nor kinetic parameters showed significant deviations from wildtype in male R6/2 mice, and the normalized force-frequency curves were almost identical. The relatively mild effects on muscle performance in soleus may come as a surprise considering the hypothesis that mitochondria dysfunction is a major factor in HD muscle pathology [10] and the fact that SOL consists mainly of mitochondria-rich type IIA and I fibers. A possible explanation for the difference is that the smaller pool of mitochondria in fast glycolytic muscle may make them more sensitive to mitochondrial dysfunction. Aging is another reported condition that leads to larger damage in fast glycolytic skeletal muscle than in slow oxidative muscle and has been attributed to higher oxidant production and lower defense against reactive oxygen species in glycolytic fibers [62].

Motor protein changes in SOL (shift from IIa to I) were confined to MyHC isoforms and to male specimen. An apparent sexual dimorphism (higher IIa/I ratio in males) as found here for WT soleus has also been reported in other cases as summarized by Schiaffino and Reggiani [37]. In our case, the sex-related differences between MyHC I and IIa expression in WT SOL muscles got balanced in the R6/2 mice. No alterations were observed in MyLC isoforms. In contrast, in the EDL, both genders showed comparable absolute levels and fractional changes in MyHC and MyLC protein expression.

### Possible causes of altered contraction kinetics in R6/2 muscle

As stated above, the higher relative expression of MyHC IIx and MyLC1f in R6/2 EDL would be consistent with slower contraction. Whether the observed changes are sufficient to fully explain the changes in kinetics remains questionable. Additional factors may contribute.

A possible important modulator is a change in the Ca^2+^-dependent control of actomyosin interaction. In addition to the alterations in twitch kinetics we observed a strong decrease in specific force (i.e. force per cross-sectional area). This is difficult to explain by atrophy or the measured shift in myosin isoforms. Decreased RNA expression for various proteins involved in the Ca^2+^ signaling cascade of muscle activation (ryanodine receptor, calsequestrin, parvalbumin, troponin) have been reported [31]. Recently, our group found a decrease in the rates of both Ca^2+^ release from the SR and removal of released Ca^2+^ in isolated interosseous muscle fibers of R6/2 mice [36]. Lower Ca^2+^ release would decrease force production, and slower Ca^2+^ removal, caused by changes in Ca^2+^ buffering or transport, should decrease the relaxation speed of twitch force and may consequently contribute to the observed left-shift in the force-frequency curve. Furthermore, reduced ryanodine receptor-mediated Ca^2+^ release may by itself lead to fast-to-slow fiber transformation [63].

Previous investigators concluded that “much of the HD muscle phenotype may result from the intrinsic expression of mHTT and its polyQ toxicity” [31]. Mutant huntingtin inclusions are found in HD skeletal muscle [25–27, 29]. However, changes in the motor axons and the nerve-muscle interface may be major contributors. Ribchester et al. [34] found denervation-like alterations and abnormalities in the neuromuscular junctions in R6/2 skeletal muscle, and Mielcarek et al. [30] interpreted their observed changes in transcription and transcriptional regulation with a denervation-like muscle phenotype affecting predominantly fast motor units. The pattern of electrical activity (number of impulses per time) is considered the main determinant of adaptive changes in skeletal muscle [37]. Chronic low frequency stimulation or motoric hyperactivity will lead to similar myosin remodeling in EDL as described here, i.e. to an increase in MyHCIIx on the expense of MyHCIIb [64] and an increase in MyLC1f on the expense of MyLC3f [65, 66]. Since integrated electrical activity of the motor axons reaching skeletal muscles can be expected to be higher in HD, this may directly impact on fiber remodeling. Such effects are difficult to distinguish from alterations originating in the muscle cells. Therefore, it remains to be resolved to which degree the skeletal muscle alterations seen in HD and corresponding animal models result from muscle intrinsic mutant huntingtin or from changes in motor neuron function and activity.

## Supporting Information

S1 Fig Relative amounts of myosin heavy chains determined at different applied protein concentrations. (A1, B1) Examples of Roti®-Blue-stained gels (A1: EDL, B1: SOL) showing protein bands from SDS-PAGE (8% gel, run time 28 h). (A2, B2) Mean values of relative amounts of the indicated MyHC isoforms in EDL and SOL, respectively. WT muscles. Error bars indicate SEM. For comparison of WT and R6/2 heavy chains (Fig 2) 5 μg protein were used.
(TIF)

S2 Fig Relative amounts of myosin light chains determined at different applied protein concentrations. (A1, B1) Examples of Roti®-Blue-stained gels (A1: EDL, B1: SOL) showing protein bands from SDS-PAGE (12% gel, run time 1.5 h). (A2, B2) Mean values of relative amounts of the indicated MyLC isoforms in EDL and SOL, respectively. WT muscles. For comparison of WT and R6/2 (Fig 4) 25 μg protein were used. Error bars indicate SEM. This and S1 Fig demonstrate that results are essentially independent of the starting values of protein concentration used in the experiments.
(TIF)
