# Supplementary figures and images for: Fast-to-Slow Transition of Skeletal Muscle Contractile Function and Corresponding Changes in Myosin Heavy and Light Chain Formation in the R6/2 Mouse Model of Huntington’s Disease
Source: PLoS One. 2016 Nov 7;11(11):e0166106. doi: 10.1371/journal.pone.0166106 (PMC5098792; doi:10.1371/journal.pone.0166106)

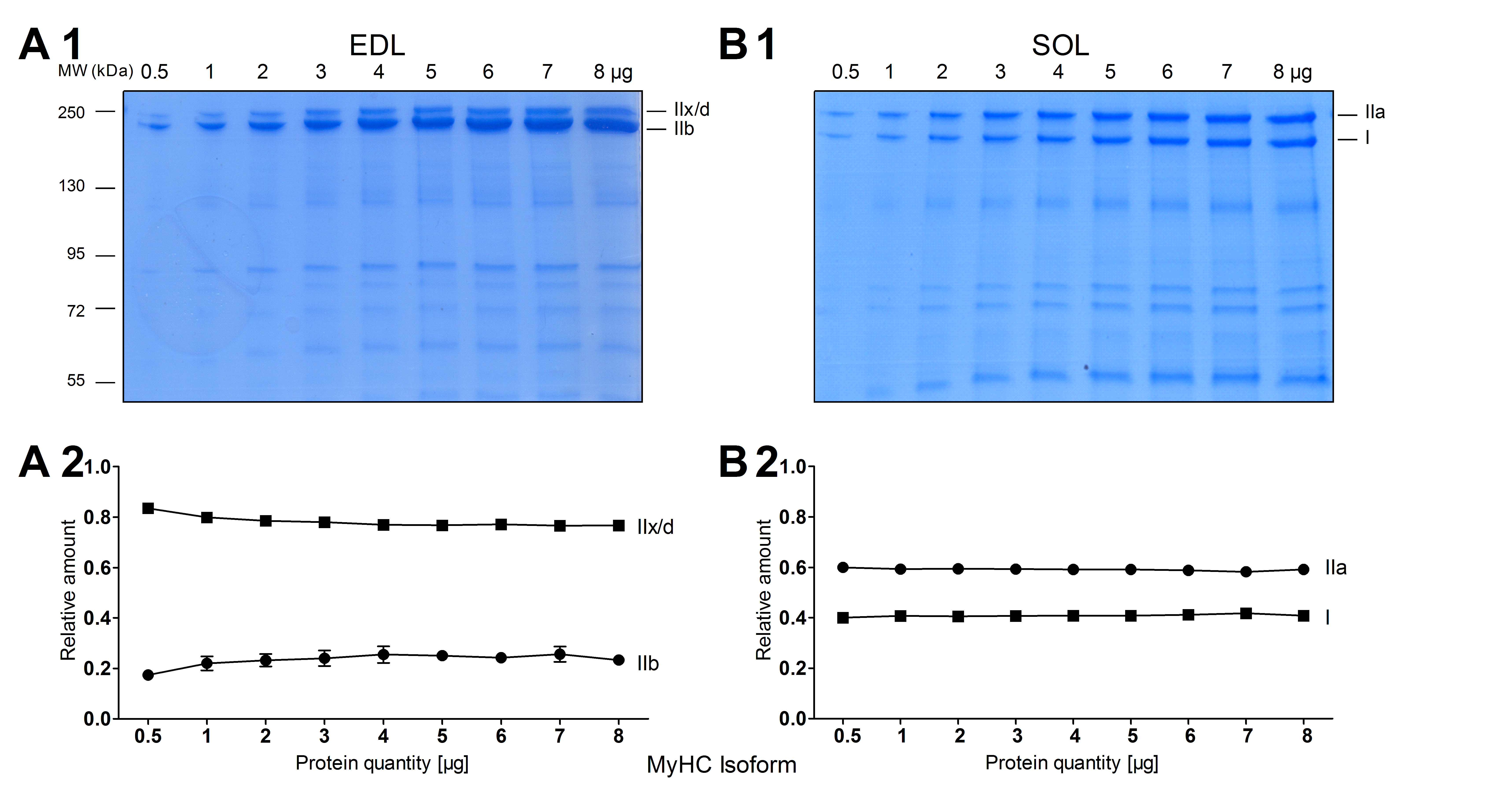

Supplement: S1 Fig — (A1, B1) Examples of Roti®-Blue-stained gels (A1: EDL, B1: SOL) showing protein bands from SDS-PAGE (8% gel, run time 28 h). (A2, B2) Mean values of relative amounts of the indicated MyHC isoforms in EDL and SOL, respectively. WT muscles. Error bars indicate SEM. For comparison of WT and R6/2 heavy chains (Fig 2) 5 μg protein were used. (TIF) [file pone.0166106.s001.tif]

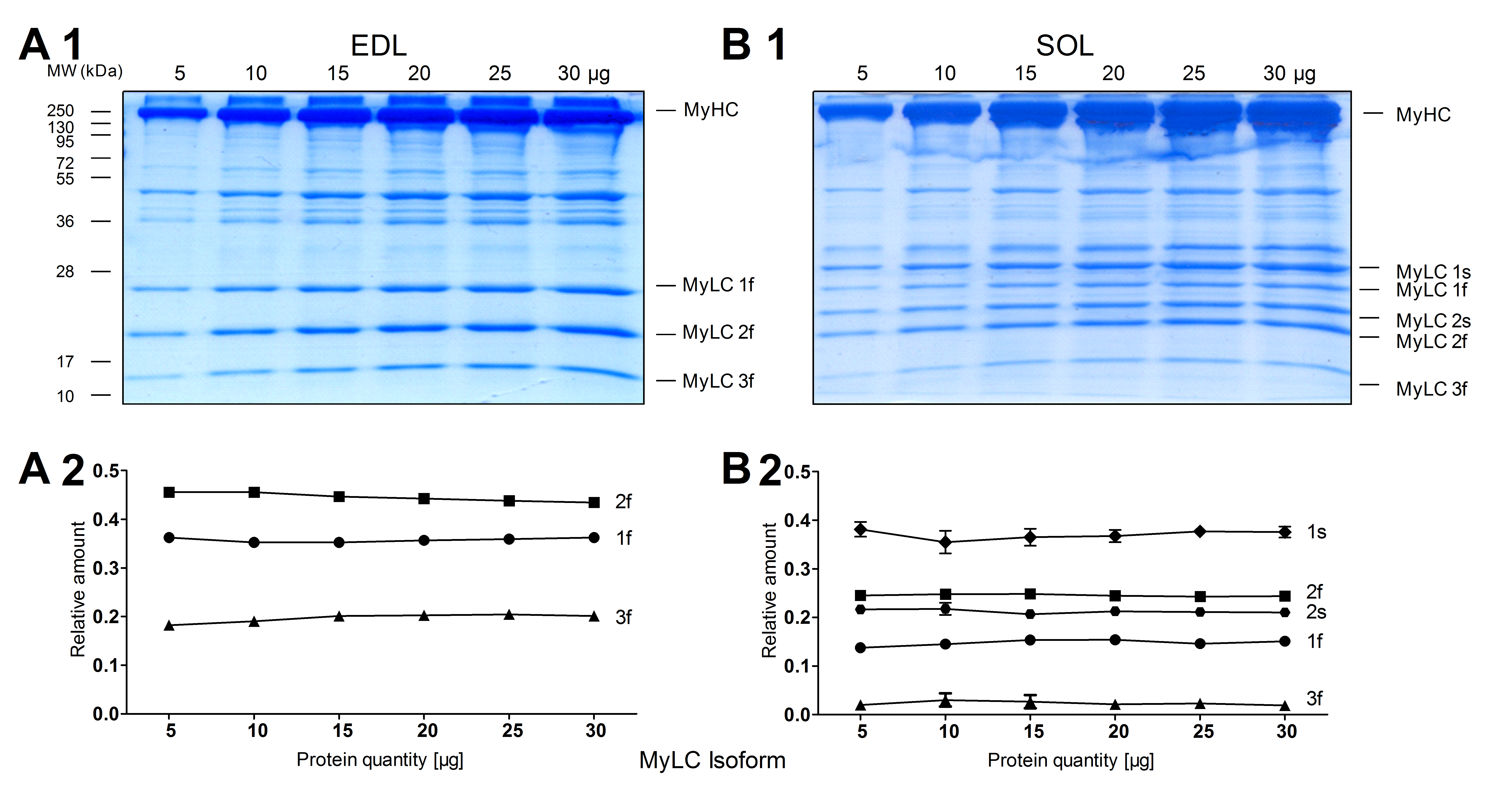

Supplement: S2 Fig — (A1, B1) Examples of Roti®-Blue-stained gels (A1: EDL, B1: SOL) showing protein bands from SDS-PAGE (12% gel, run time 1.5 h). (A2, B2) Mean values of relative amounts of the indicated MyLC isoforms in EDL and SOL, respectively. WT muscles. For comparison of WT and R6/2 (Fig 4) 25 μg protein were used. Error bars indicate SEM. This and S1 Fig demonstrate that results are essentially independent of the starting values of protein concentration used in the experiments. (TIF) [file pone.0166106.s002.tif]
